# Comparison of plastid proteomes points towards a higher plastidial redox turnover in vascular tissues than in mesophyll cells

**DOI:** 10.1093/jxb/erad133

**Published:** 2023-04-07

**Authors:** Clément Boussardon, Chris Carrie, Olivier Keech

**Affiliations:** Department of Plant Physiology, Umeå Plant Science Centre, Umeå University, S-90187 Umeå, Sweden; School of Biological Sciences, University of Auckland, 3A Symonds St, Auckland,1142, New Zealand; Department of Plant Physiology, Umeå Plant Science Centre, Umeå University, S-90187 Umeå, Sweden; University of Helsinki, Finland

**Keywords:** Biotin–streptavidin interaction, cyclic electron flow, plastids, plastoglobuli, proteomics, vascular and mesophyll cells

## Abstract

Plastids are complex organelles that vary in size and function depending on the cell type. Accordingly, they can be referred to as amyloplasts, chloroplasts, chromoplasts, etioplasts, or proplasts, to only cite a few. Over the past decades, methods based on density gradients and differential centrifugation have been extensively used for the purification of plastids. However, these methods need large amounts of starting material, and hardly provide a tissue-specific resolution. Here, we applied our IPTACT (Isolation of Plastids TAgged in specific Cell Types) method, which involves the biotinylation of plastids *in vivo* using one-shot transgenic lines expressing the *Translocon of the Outer Membrane 64* (*TOC64*) gene coupled with a biotin ligase receptor particle and the *BirA* biotin ligase, to isolate plastids from mesophyll and companion cells of Arabidopsis using tissue specific *pCAB3* and *pSUC2* promoters, respectively. Subsequently, a proteome profiling was performed, which allowed the identification of 1672 proteins, among which 1342 were predicted to be plastidial, and 705 were fully confirmed according to the SUBA5 database. Interestingly, although 92% of plastidial proteins were equally distributed between the two tissues, we observed an accumulation of proteins associated with jasmonic acid biosynthesis, plastoglobuli (e.g. NAD(P)H dehydrogenase C1, vitamin E deficient 1, plastoglobulin of 34 kDa, ABC1-like kinase 1) and cyclic electron flow in plastids originating from vascular tissue. Besides demonstrating the technical feasibility of isolating plastids in a tissue-specific manner, our work provides strong evidence that plastids from vascular tissue have a higher redox turnover to ensure optimal functioning, notably under high solute strength as encountered in vascular cells.

## Teaser

Using a novel method (IPTACT) to isolate plastids, it is shown that plastids from Arabidopsis vascular tissues have a higher redox turnover than those from mesophyll cells.

## Introduction

The plastid is one of the three genome-containing compartments of a plant cell, together with the nucleus and the mitochondrion. The chloroplast is undoubtedly the most studied representative of the plastid family, but it only represents a fraction of the functional plastids found in plants. Indeed, plastids can diverge in size, shape, internal structure, and metabolic function depending on the cell type to which they belong. Accordingly, a dedicated nomenclature has been adopted with amyloplasts, chromoplasts, dessicoplasts, elaioplasts, etioplasts, gerontoplasts, leucoplasts, phenyloplasts, proplastids, proteinoplasts, and even xyloplasts, as recently proposed by Pinard and Mizrachi (2018).

So far, methods to isolate pure and intact plastids have mostly been based on density gradients coupled to differential centrifugation steps. These were successfully employed to isolate plastids from different organs and for diverse species. For instance, several studies reported the isolation of chloroplasts from leaves of Arabidopsis (Kleffmann *et al.*, 2004; Seigneurin-Berny *et al.*, 2008), *Zea mays* (maize; Huang *et al.*, 2013), *Triticum aestivum* (wheat; Balmer *et al.*, 2006), *Oryza sativa* (rice; von Zychlinski et al., 2005), *Pisum sativum* (pea; Bayer *et al.*, 2011; Simm *et al.*, 2013), and *Medicago truncatula* (medicago; Simm *et al.*, 2013), to cite only a few. Similarly, plastids from Arabidopsis roots have also been obtained, allowing the identification of a specific subset of proteins that suggested that root plastids are more versed in nitrogen/sulfur metabolism and amino acid biosynthesis than in the storage of sugars (Grabsztunowicz *et al.*, 2020). Chromoplasts from *Solanum lycopersicum* (tomato; Suzuki *et al.*, 2015) and *Capsicum annuum* (bell pepper; Rodiger *et al.*, 2021) fruits were obtained using density gradients (Nycodenz or Percoll, respectively). This work notably led to the identification of several types of metabolic regulation associated with chromoplast differentiation. Using the same species, i.e. *Solanum lycopersicum* and *Capsicum annuum*, an alternative method based on a sucrose gradient has also been reported for isolating intact chromoplasts at different developmental stages (Barsan *et al.*, 2017).

While these studies have often provided very valuable data on plastid functions in the selected organ, the experimental strategies could not offer a deeper resolution. For this reason, alternative studies have proposed a tissue-specific approach, including paradermal sectioning (Terashima and Saeki, 1983; Terashima *et al.*, 1986), differential grinding (Majeran *et al.*, 2005), the use of enhancer trap lines (Truernit and Hibberd, 2007), or fluorescence-activated cell sorting (Beltran *et al.*, 2018). These methods have helped to define the role of plastids in particular cell types. For example, in maize, the proteomic comparison of chloroplasts originating from bundle sheath and mesophyll cells revealed a disparity in the accumulation of starch and lipid and in isoprenoid biosynthesis enzymes as well as nitrogen and sulfur import enzymes (Majeran *et al.*, 2005). Beltran *et al.* (2018) showed in Arabidopsis that certain stress-related proteins that were previously assigned to mesophyll chloroplasts were actually mostly localized to the epidermal and vascular parenchyma cell plastids (named ‘sensory’ plastids), and subsequently proposed that retrograde signaling could differ between the different types of tissue. This shows that plastids originating from different cell types have specific functions and undergo different levels of regulation.

Although these methods are of high quality, they remain tedious in terms of both time and reliability. Recently, an elegant alternative method to obtain intact and pure organelles was proposed with the SpySystem, which allowed for the isolation of mitochondria and plastids (Lang *et al.*, 2020). Briefly, this method relies on the covalent interaction between genetically encoded SpyTag fusion constructs containing OEP7 (plastid) or NtHXK1 (mitochondria) targeting sequences under the control of a 35S promoter and SpyCatcher-coated beads. In the same vein, we published an original method, called IMTACT, to isolate mitochondria from Arabidopsis with tissue-specific resolution (Boussardon *et al.*, 2020; Boussardon and Keech, 2022). Our method is based on an *in vivo* biotinylation of tagged OM64 proteins, and the subsequent isolation of the streptavidin beads–mitochondria complex using a magnetic field. Recently, we adapted this method to isolate plastids in a tissue-specific manner. The method, termed IPTACT (for Isolation of Plastids TAgged in specific Cell Types), relies on a similar strategy, i.e. the biotinylation of plastids using a one-shot transgenic line expressing the *Translocon of the Outer Membrane 64* (*TOC64*) gene coupled with a biotin ligase receptor particle and the *BirA* biotin ligase. Here, after demonstrating the efficiency and reliability of the method for isolating plastids from mesophyll and vascular cells, we compared the proteomes of these plastids to gain insight into the physiological function of plastids from vascular tissues. We discovered that although both proteomes were globally similar, plastids from vascular tissues had an accumulation of proteins associated with plastoglobuli and with jasmonate (JA) metabolism as well as an enhanced redox turnover.

## Materials and methods

### Plant material and growth conditions

Arabidopsis Columbia-0 (Col-0) wild type (WT) and transgenic lines plants were grown under short-day photoperiod (SD: light 8 h at 22 °C, dark 16 h at 17 °C) at 65% relative humidity and 180 μmol m^−2^ s^−1^ photosynthetically active radiation (PAR) or long-day photoperiod (LD: light 16 h at 22 °C, dark 8 h at 17 °C) at 65% relative humidity and 150 μmol m^−2^ s^−1^ PAR on a 3:1 soil:vermiculite mixture.

LD growth conditions were used for propagation of new generations and *in vitro* plantlet selection on selective media and SD growth conditions were used for the single plant IPTACT assays.

Seeds were systematically sterilized with 70% ethanol for 10 min, followed by 10 min in 56% ethanol. Once ethanol was discarded, seeds were air-dried for 1 h and stratified at 4 °C in 0.1% agarose for 3 d.

### Plasmid construction and *Agrobacterium*-mediated transformation

Golden Gate constructs were made according to the protocols outlined in Binder *et al.* (2014) and Chiasson *et al.* (2019). The *SUC2* (AT1G22710, 1465 bp, named *pSUC2*) and *CAB3* (AT1G29910, 1537 bp, named *pCAB3*) promoters were amplified by PCR (adding *Bsa*I sites in 5ʹ and 3ʹ) to create LI PCR products (Supplementary Table S1). The *ccdB* cassette was replaced by *pSUC2* or *pCAB3* using a LI/LIII ligation/cut reaction (100 ng LIII vector, 5 µl of purified PCR product, 1.5 µl 10× Buffer G, 1.5 µl 10 mM ATP, 0.75 µl *Bsa*I 20 U/µl, 0.75 µl T4 DNA ligase 5 U/µl, H_2_O to 15 µl).

Plasmids were used for *Agrobacterium tumefaciens* (GV3101::pMP90, pSOUP) transformation of Col-0 using the floral dip method (Clough and Bent, 1998). *In vitro* selections of T_1_ plantlets were made on ½ MS + 0.1% sucrose medium containing Basta (10 μg/ml).

### IPTACT procedure

A step-by-step protocol can be found in Boussardon and Keech (2023). Briefly, all steps were performed in a cold room (at 4 °C), on ice, with pre-cooled materials and under green light. The day prior to extraction, plants were placed in complete darkness to prevent starch formation. About 1 g of leaves was ground in 20 ml of isolation buffer (0.3 M d-sorbitol, 5 mM MgCl_2_, 5 mM EGTA, 5 mM EDTA, 20 mM Hepes, 10 mM NaHCO_3_, pH adjusted with KOH to 8; see Aronsson and Jarvis, 2002) using a polytron (speed 4 out of 11, 2 s pulse, repeated three times). Homogenate was filtered through a pre-wet double layer of Miracloth. Debris retained in the Miracloth was homogenized with a Polytron in 20 ml of isolation buffer, filtered again, and added to the first extract. The homogenate was then centrifuged at 1000 *g* for 5 min at 4 °C. The resulting pellet was resuspended in 20 ml of HEPES–MgSO_4_–sorbitol buffer (HMS; 0.3 M d-sorbitol, 3 mM MgSO_4_, 50 mM Hepes, pH adjusted with KOH to 7.6; see Aronsson and Jarvis, 2002) using a brush. The resulting homogenate was centrifuged one more time at 1000 *g* for 5 min at 4 °C. The pellet was then resuspended in 5 ml of HMS buffer to obtain a crude plastid homogenate, referred to as crude extract.

While the centrifugations operated, 2.8 µm magnetic beads (Thermo Fisher Scientific Dynabead M-280 Streptavidin, cat. no. 11205D) were prepared according to the manufacturer’s instructions. Briefly, beads were vortexed for 30 s, and 30 µl of beads was transferred in a 2 ml Eppendorf tube. One volume of HMS buffer was added, and beads were separated using a magnet (Miltenyi Biotec, MiniMACS Separator). The supernatant was discarded, and beads were washed three times with HMS buffer. Finally, beads were resuspended in one bead volume of HMS buffer.

The crude extract was mixed with the magnetic beads and gently inverted for 1 min to avoid crushing the plastids. Then, the beads were separated using a magnet for 2 min and the supernatant was discarded. The beads were washed four times using 800 µl of HMS buffer (resuspended by inverting tube) and were finally resuspended in 80 µl HMS buffer. Proteins were collected using a hypotonic buffer to rupture the plastids (50 mM HEPES–KOH pH 7.5, 5 mM MgCl_2_, 10 mM NaF; Bag *et al.*, 2021). The protein suspension was then used for protein quantification and immunoblotting.

### Microscopy

#### Confocal imaging

Fluorescence of the TOC64–enhanced green fluorescent protein (eGFP)–biotin ligase recognition peptide (BLRP) protein was observed with a confocal microscope (Zeiss, LSM780) using Zeiss Zen software. Signals were detected according to the following excitation/emission wavelengths: eGFP (488 nm/495–535 nm) and chloroplast autofluoresence (633 nm/660–720 nm). Pictures were analysed using ImageJ software (https://imagej.nih.gov/ij/).

#### Scanning electron microscopy

For scanning electron microscopy (SEM), samples were fixed, dispersed, and sedimented onto glass coverslips, subsequently dehydrated in a graded ethanol series, critical point dried and coated with 2 nm iridium. The morphology was analysed by field-emission scanning electron microscopy (FESEM; Carl Zeiss Merlin) using a secondary electron detector at accelerating voltage of 2–4 kV and probe current of 100 pA. Elemental distribution was performed using an energy dispersive X-ray spectrometer (EDS; Oxford Instruments X-Max 80 mm^2^) at an accelerating voltage of 10 kV and probe current of 300 pA.

### SDS-PAGE immunoblot assay

For immunoblot analysis, protein quantification was performed with a Bradford protein assay (Bio-Rad). Crude extracts and bead-bound isolated plastids were mixed with Laemmli sampling buffer (Bio-Rad) supplemented with 200 mM dithiothreitol and incubated at 95 °C for 10 min before separating the protein mixtures on reducing 10 or 12% polyacrylamide gels.

After migration at 100 V, proteins were transferred for 1 h at 270 mA onto a 0.45 µm nitrocellulose membrane. Membranes were blocked with 5% milk in Tris-buffered saline–Tween 20 (TBS-T) for 1 h followed by an overnight incubation at 4 °C with specific polyclonal primary antibodies anti-Rubisco large subunit (RbcL; 1/2000, Agrisera, AS03 037), anti-LHCII type I chlorophyll *a*/*b*-binding protein (LHCB1; 1/1000, Agrisera, AS01 004), anti-translocon of the outer envelope membrane of chloroplasts 34 protein (TOC34; 1/2000, Agrisera, AS07 235), anti-Glycine Decarboxylase Complex (GDC)-H subunits (1/1000, Agrisera, AS05 074), anti-isocitrate dehydrogenase (IDH; 1/1000, Agrisera, AS06 203A), anti-histone3 (HIS3; 1/5000, Agrisera, AS10 710), anti-SEC12 (1/3000; Zheng *et al.*, 2002), anti-catalase (1/1000, Agrisera, AS09 501), anti-UDP-glucose pyrophosphorylase (UGPase; 1/1000, Agrisera, AS05 086), or anti-γ-tonoplast intrinsic protein (TIP1;1; 1/3000; Rojo *et al.*, 2003) diluted in 2% milk in TBS-T. After 1 h incubation at room temperature with a goat anti-rabbit or rabbit anti-chicken (for TIP1;1) secondary antibodies conjugated to horseradish peroxidase (1/10000 in 2% milk in TBS-T, Agrisera, AS09 602), visualization was carried out using a chemiluminescence kit (Agrisera ECL kit bright; AS16 ECL-N-100) and signals were detected using the Azure c600 Western Blot Imaging system (Azure biosystems). Exposure time was 30 s.

### Tissue specific proteomic analysis

#### Samples preparation for MS/MS

Col-0 *pCAB3::* and *pSUC2::TOC64-eGFP-BLRP–UBQ10-BirA* plants were grown in SD for 7 weeks. Leaves from single plants were collected in the early morning and the IPTACT procedure was applied. For each line, four biological replicates were produced. After Bradford quantification, 12–20 µg (*pSUC2*) or 20–26 µg (*pCAB3*) of protein was aliquoted, frozen using liquid nitrogen, and stored at −80 °C. For each line, an equimolar mixture of the four replicates was created to obtain a fifth replicate. Plastids collected from mesophyll cells and companion cells were lysed by 4% SDS lysis buffer and prepared for mass spectrometry analysis using a modified version of the SP3 protein clean-up and digestion protocol (Moggridge *et al.*, 2018). Peptides were labelled with TMT10plex reagent according to the manufacturer’s protocol (Thermo Fisher Scientific, A37725). In brief, 12–26 µg protein from each sample was alkylated with 4 mM chloroacetamide. Sera-Mag SP3 bead mix (20 µl) was transferred into the protein sample together with 100% acetonitrile to a final concentration of 70%. The mix was incubated under rotation at room temperature for 18 min. The mix was placed on a magnetic rack and the supernatant was discarded, followed by two washes with 70% ethanol and one with 100% acetonitrile. The beads–protein mixture was reconstituted in 100 µl LysC buffer (0.5 M Urea, 50 mM HEPES pH 7.6 and 1:50 enzyme (LysC) to protein ratio) and incubated overnight. Finally, trypsin was added in 1:50 enzyme to protein ratio in 100 µl 50 mM HEPES pH 7.6 and incubated overnight. The peptides were eluted from the mixture after placing the mixture on a magnetic rack, followed by peptide concentration measurement (MicroBCA Assay, Thermo Fisher Scientific). Ten micrograms of peptides from each sample was labeled with isobaric tandem mass tag (TMT) tags. Before labelling, samples were pH adjusted using triethylammonium bicarbonate pH 8.5 (100 mM final conc.). Labelling efficiency was determined by LC-MS/MS before pooling of samples. Sample clean-up was performed by solid phase extraction (SPE strata-X-C, Phenomenex) and purified samples were dried in a SpeedVac. An aliquot of approximately 10 µg was suspended in LC mobile phase A and 4 µg was injected into the LC-MS/MS system.

#### MS/MS procedure

LC–electrospray ionization–MS/MS Q-Exactive Online LC-MS was performed using a Dionex UltiMate 3000 RSLCnano System coupled to a Thermo Scientific High Field Q Exactive mass spectrometer. Four microliters was injected from each sample. Samples were trapped on a C18 guard desalting column (Acclaim PepMap 100, 75 µm×2 cm, nanoViper, C18, 5 µm, 100 Å), and separated on a 50 cm-long C18 column (Easy spray PepMap RSLC, C18, 2 µm, 100 Å, 75 µm×15 cm). The nanocapillary solvent A was 95% water, 5% dimethyl sulfoxide (DMSO), 0.1% formic acid; and solvent B was 5% water, 5% DMSO, 95% acetonitrile, 0.1% formic acid. At a constant flow of 0.25 μl min^−1^, the curved gradient went from 6% B up to 40% B in 240 min, followed by a steep increase to 100% B in 5 min. Fourier transform MS master scans with 60 000 resolution (and mass range 300–1500 *m*/*z*) were followed by data-dependent MS/MS (30 000 resolution) on the top five ions using higher energy collision dissociation at 30% normalized collision energy. Precursors were isolated with a 2 *m*/*z* window. Automatic gain control targets were 1e6 for MS1 and 1e5 for MS2. Maximum injection times were 100 ms for MS1 and MS2. The entire duty cycle lasted ~2.5 s. Dynamic exclusion was used with 60 s duration. Precursors with unassigned charge state or charge state 1 were excluded. An underfill ratio of 1% was used.

#### Peptide and protein identification

Orbitrap raw MS/MS files were converted to mzML format using msConvert from the ProteoWizard tool suite (Holman *et al.*, 2014). Spectra were then searched using MSGF+ (v10072) (Kim and Pevzner, 2014) and Percolator (v2.08) (Granholm *et al.*, 2014). All searches were done against the Arabidopsis Columbia protein subset of Uniprot in the Nextflow platform (https://github.com/lehtiolab/ddamsproteomics, v1.5) built using the workflow tool Nextflow (v19.04.0). MSGF+ settings included precursor mass tolerance of 10 ppm, fully tryptic peptides, maximum peptide length of 50 amino acids and a maximum charge of 6. Fixed modifications were TMT10 plex on lysines and peptide N-termini, and carbamidomethylation on cysteine residues; a variable modification was used for oxidation on methionine residues. Quantification of TMT10 plex reporter ions was done using OpenMS project’s IsobaricAnalyzer (v2.0) (Sturm *et al.*, 2008). Peptide–spectrum matches (PSMs) found at 1% false discovery rate (FDR) were used to infer gene identities. Protein quantification by TMT10 plex reporter ions was calculated using TMT PSM ratios to the entire sample set (all 10 TMT channels) and normalized to the sample median. The median PSM TMT reporter ratio from peptides unique to a gene symbol was used for quantification. Protein FDRs were calculated using the picked-FDR method using gene symbols as protein groups and limited to 1% FDR (Savitski *et al.*, 2015). Mass spectrometry analysis was performed by the Clinical Proteomics Mass Spectrometry facility, Karolinska Institutet/Karolinska University Hospital/Science for Life Laboratory.

#### Cellular localization

The SUBA5 database was used to identify the cellular localization of proteins (Hooper *et al.*, 2017; https://suba.live/). For GO analysis, enriched GO terms were obtained from g:Profiler (Raudvere *et al.*, 2019; organism=*Arabidopsis thaliana* TAIR10 (EnsemblPlants); threshold=Benjamini–Hochberg FDR; *P*<0.01) and the biological network was created using Revigo (Supek *et al.*, 2011; list=medium (0.7); GO terms=*P*-value; species=*Arabidopsis thaliana* (NCBI:txid3702); semantic similarity=SimRel).

## Results

### Generation of CAB3 and SUC2 IPTACT lines

**Fig. 1.**
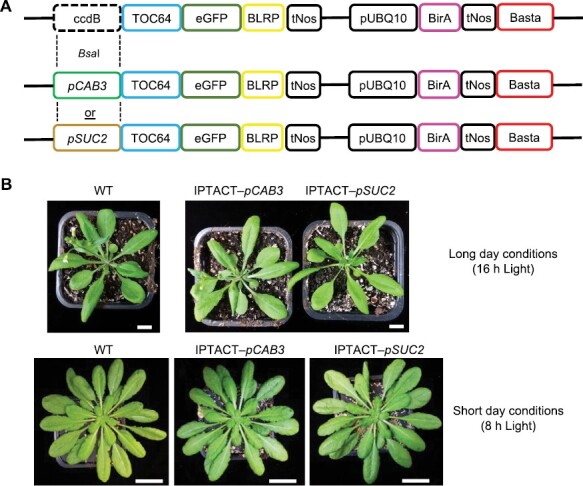
One-shot construct for tissue-specific transformations and growth phenotype. (A) *BirA* expression is driven by a constitutive *UBQ10* promoter. The *TOC64* cDNA full length form (*TOC64*) was fused with *eGFP* and *BLRP*. Instead of a promoter, a *ccdB* cassette has been inserted in the 5ʹ end of the *TOC64* translational fusion. The *ccdB* cassette can be replaced using Golden Gate cloning by the desired promoter, thus allowing the modulation of TOC64 expression (tissue- or development-specific). The selection marker (Basta) was inserted at the 3ʹ end of the construct. (B) Growth phenotype of Col-0 WT and stable transformed plants after 4 weeks under long-day growth conditions (top; scale bars: 1 cm) or 7 weeks under short-day growth conditions (bottom; scale bars: 2 cm).

We first generated a set of Golden Gate plasmids containing two expression cassettes. The first expression cassette contains the N-terminal anchored envelope *TOC64* cDNA sequence in-frame with an *eGFP* reporter and a biotin ligase recognition peptide (*BLRP*) gene. A *ccdB* gene, used for negative selection during cloning reactions, flanked by *Bsa*I restriction sites, was placed upstream of *TOC64*. The second expression cassette contained the biotin ligase (*BirA*) gene under the control of the *UBQ10* promoter and the *NOS* terminator. The selection marker (Basta) was inserted at the 3ʹ end of the construct (Fig. 1A). Note that additional constructs with two other selection markers (hygromycin and kanamycin) have been generated (see ‘Materials and methods’; Boussardon and Keech, 2023).

To obtain the tissue-specific IPTACT lines, the mesophyll-specific promoter of the *CHLOROPHYLL A/B BINDING PROTEIN 3* (*CAB3*) gene (Susek *et al.*, 1993) and the companion cell-specific promoter of the *ARABIDOPSIS THALIANA SUCROSE-PROTON SYMPORTER 2* (*SUC2*) gene (Truernit and Sauer, 1995) were PCR-amplified and flanked with *Bsa*I restriction sites. The promoter amplicons were used to replace the *ccdB* negative selection gene and to drive the expression of the TOC64 cassette in either the mesophyll cells or companion cells.

Plants transformed with either the *pSUC2::TOC64-eGFP-BLRP* or *pCAB3::TOC64-eGFP-BLRP* transgenes did not show any macroscopic phenotypes when compared with WT Col-0 (Fig. 1B). Also, the chlorophyll content is similar between the transgenic lines and the WT Col-0 (Supplementary Fig. S1). Thus, we concluded that expression of the transgene does not affect plastid functions, and consequently plant growth and development.

**Fig. 2.**
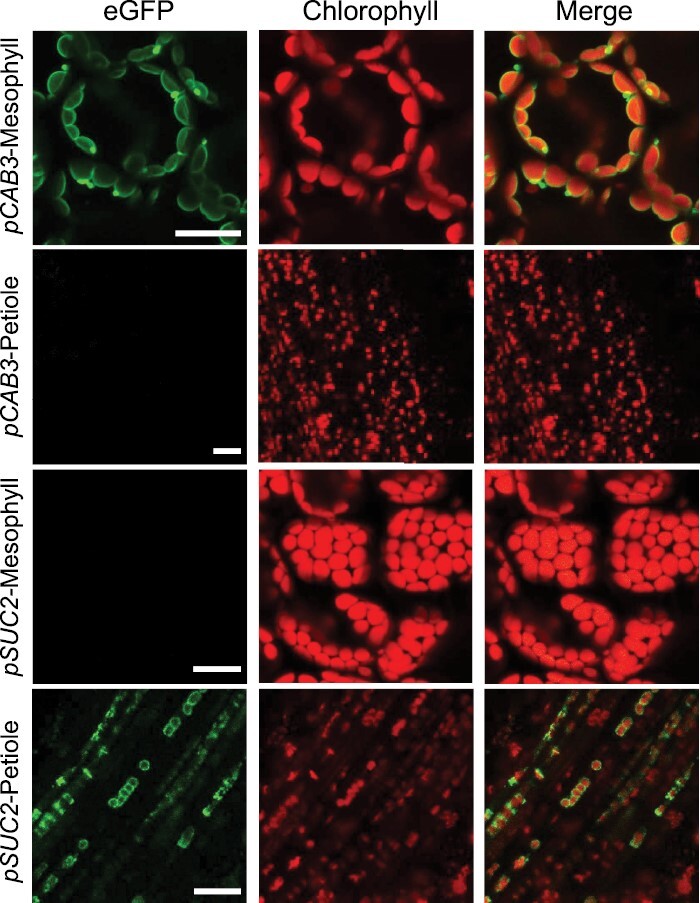
*In vivo* visualization of tagged plastids. Fluorescence images of Arabidopsis mesophyll and petiole expressing the TOC64–eGFP–BLRP fusion protein. Leaves from stable transformation lines were observed by confocal microscopy (×40 magnification, water immersion), and co-localization was assessed using chlorophyll autofluorescence. TOC64–eGFP–BLRP construct expression is driven by either the *pCAB3* mesophyll specific promoter or the *pSUC2* companion cell-specific promoter. Scale bars: 20 µm.

The tissue-specific localization of TOC64–eGFP–BLRP was assessed by confocal laser microscopy (Fig. 2). The emission of the eGFP was confirmed in the mesophyll for lines expressing *TOC64* under the control of the *pCAB3* promoter. The signal co-localized with the plastid autofluorescence confirming that the fusion protein was specific to plastids. As expected, the eGFP signal was seen to be encircling the chlorophyll autofluorescence indicating an envelop membrane localization of the fusion protein as previously described for OEP7 (Farmaki *et al.*, 2007; Machettira *et al.*, 2011). For the *pSUC2::TOC64-eGFP-BLRP* construct, fluorescent plastids were observed aligned in the veins, a pattern characteristic of the companion cell plastids (Cayla *et al.*, 2015). A protrusion in the plastid envelope was observed in some *pCAB3* lines. We assume that this is related to the increased concentration of the envelope protein as it was previously observed in TIC20 and OEP7 overexpression lines (Machettira *et al.*, 2011). These data demonstrated that (i) the TOC64–eGFP–BLRP translational fusions are localized to the outer envelope of plastids *in vivo* and (ii) promoters driving the transgene expression give the correct tissue specificity.

### Purification of biotinylated plastids and quality control assessment of the IPTACT method

We sampled leaves from short-day-grown T_3_ homozygous Col-0 plants containing the *TOC64-eGFP-BLRP* transgene under the control of either the *pCAB3* or *pSUC2* promoter. Plastids were isolated using a procedure named IPTACT, a method using the same principle as INTACT for nuclei (Deal and Henikoff, 2011) and IMTACT for mitochondria (Boussardon *et al.*, 2020). A plastid-enriched extract was obtained according to the first steps of the method published by Aronsson and Jarvis (2002). Briefly, 1 g of tissue was collected and homogenized in an isolation buffer using a 13-mm Polytron homogenizer. The homogenate was filtered through a double layer of Miracloth and centrifuged to collect a plastid-enriched fraction. This extract was incubated for 1 min with streptavidin magnetic beads, as 1 min was sufficient for the IMTACT procedure (Boussardon *et al.*, 2020). As BirA biotinylates BLRP, plastids bearing the translational fusion interact with the streptavidin beads leading to a green pellet. After washing, the plastids were resuspended in a HMS buffer as described in Aronsson and Jarvis (2002). This buffer was shown to be more efficient than the HS buffer (lacking 3 mM MgSO_4_) for protein import, suggesting a better physiological state of the plastid envelope. We estimated that between 30 and 40 µg of plastid proteins was isolated from the *pCAB3* lines (Supplementary Fig. S2; Supplementary Table S2), while only 10–20 µg of plastid proteins was extracted from *pSUC2* lines. This most likely represents the abundance of the two different cell types within 1 g of leaf tissue.

**Fig. 3.**
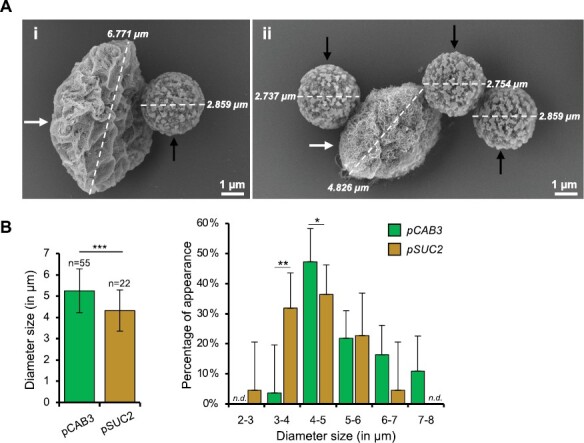
Shape and size distribution of plastids isolated by IPTACT. (A) Scanning electron microscopy (SEM). (Ai) A magnetic bead bound to a single mesophyll cell chloroplast. (Aii) Two beads bound to a vascular cell plastid. A black arrow indicates a bead while a white arrow indicates of plastid. Scale bars: 1 µm. Bead and plastid size were calculated and are shown on the figure. Note that the ‘shrivelled aspect’ of the plastid in SEM results from the electron beam when scanning. (B) Plastid size. Left, average size of plastids isolated from *pCAB3* and *pSUC2* IPTACT lines (*t*-test shown; two sided; ***P<0.001). Right, distribution of the plastidial population by diameter (a multiple two sided *t*-test showed statistically significant differences at *P<0.05 and **P<0.01; n.d., not detected).

An aliquot of the resuspended plastid–bead mixture was observed using SEM to check the plastid–beads interaction as well as mesophyll and companion cell plastid size. In contrast to the isolation procedure of plant mitochondria, we used beads with a size of approximately 2.8 µm as indicated by the provider (Fig. 3A; Boussardon and Keech, 2023). In general, plastids had an ellipsoid shape with a diameter ranging from 3 to 8 µm. Differences in size were observed between the plastids isolated from mesophyll and phloem cells (Fig. 3B). Interestingly, while the plastids from mesophyll cells were ca. 5–8 µm in diameter, plastids from phloem cells were a fraction smaller, ca. 3–6 µm. This is in agreement with previous observations (Cayla *et al.*, 2015), and suggests that the pSUC2 IPTACT lines allowed the isolation of plastids from both the companion cells and the phloem parenchyma cells. That said, at this stage we cannot rule out that plastids from other elements of phloem, such as sieve elements, are also present in our preparation, and so, we cannot exclude either that plastids from other elements of phloem are present. Therefore, to avoid any confusion, plastids isolated from pSUC2 lines are hereafter named vascular cell plastids.

**Fig. 4.**
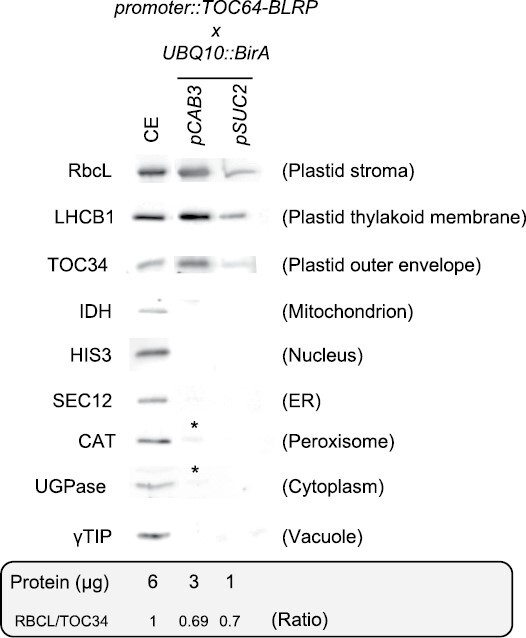
Purity control of plastids isolated by IPTACT. Ten microliters of crude extract (CE) and 10 µl of purified biotinylated plastids from shoots were immunoblotted with anti-RbcL, anti-LHCB1 and anti-TOC34 to observe plastids enrichment. Anti-IDH, anti-HIS3, anti-SEC12, anti-CAT, anti-UGPase, and anti-TIP1;1 antibodies were used to check contamination in mitochondrial, nuclear, endoplasmic reticulum (ER), peroxisomal, cytoplasmic and vacuolar proteins, respectively. Asterisks represent traces of peroxisomal and cytoplasmic contamination. Total protein content in 10 µl as well as RbcL to TOC34 ratio are also given as an estimate of the integrity of the plastids after the procedure of isolation. Images of the full membranes are available in Supplementary Fig. S3; the lane for crude extract acting as control was the same as in Boussardon and Keech (2023).

These extracts were then tested by immunoblotting on a 10 µl volume basis to check for the presence of intact plastids. To this end, immunoblot assays using the Rubisco large subunit (RbcL), LHCII type I chlorophyll *a*/*b*-binding protein (LHCB1), and translocon of the outer envelope membrane of chloroplasts 34 protein (TOC34) were performed to check the stroma, thylakoid membrane, and the outer envelope of plastids, respectively (Fig. 4; Supplementary Fig. S3). A similar signal for all three proteins was observed for the crude and *pCAB3* samples. As expected for the same volume, the *pSUC2* signal was lower than for *pCAB3*. As a rough estimation of the integrity of the isolated plastids, the stroma/envelope (RbcL/TOC34) signal ratio was used. The ratio RbcL/TOC34 was set at 1 in the crude extract, and this ratio appeared slightly ­affected by the IPTACT procedure with both *CAB3* and *SUC2* driven lines (Fig. 4). This suggests that about 70% of the plastids remained unaffected by the isolation.

Further immunoblots were performed to check whether the IPTACT plastidial preparations were contaminated by mitochondria with antibodies directed against the mitochondrial isocitrate dehydrogenase protein (IDH), by nuclei with anti-histone 3 (HIS3), by the endoplasmic reticulum with anti-SEC12 (SEC12), by peroxisomes with anti-catalase (CAT), by the cytosol with anti-UDP-glucose pyrophosphorylase (UGPase) or by vacuoles with anti-γ-tonoplast intrinsic protein (TIP1;1) (Fig. 4; Supplementary Fig. S3). Mitochondrial, nuclear, vacuolar, and endoplasmic reticulum contaminations were only observed in the crude extracts while the samples issued from IPTACT were free from contaminants. However, a slight but recurrent contamination by peroxisomal and cytosolic proteins was observed in the *pCAB3* samples. Taken together, these results show that the IPTACT procedure provided intact plastids in a rapid and tissue-specific manner with minimal contamination from other cellular compartments.

### Proteomic profiling of vascular and mesophyll cells plastids

**Fig. 5.**
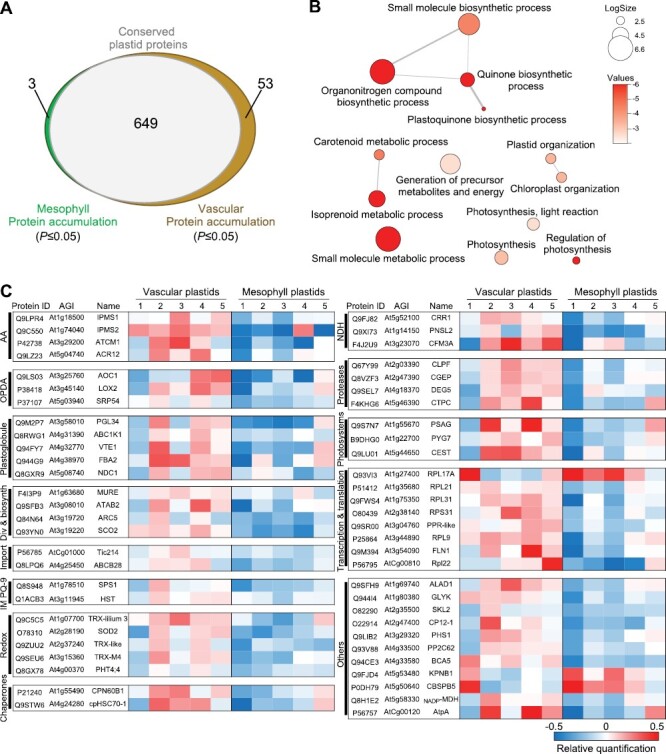
Proteomic analysis of vascular and mesophyll cell plastids. (A) Venn diagram. Differential plastid protein (SUBA5 consensus) accumulation analysis was carried out comparing mesophyll (green) and vascular (brown) cells plastids. (B) Biological network of enriched GO terms (Biological Process) from significantly (*P*≤0.05) more accumulated proteins. Enriched GO terms were obtained from g:Profiler (Raudvere *et al.*, 2019; Threshold=Benjamini–Hochberg FDR; *P*<0.01) and the biological network was created using Revigo (Supek *et al.*, 2011; values=log_10_*P*-value). LogSize corresponds to the number of annotations for the GO term ID. (C) Plastidial proteins differentially accumulated in vascular and mesophyll tissues. The 56 proteins were identified by Student’s *t*-test (two sided) comparing vascular and mesophyll protein replicates (*P*≤0.05). Protein ID, AGI code and common gene name are listed. Relative quantification of protein for each biological replicate (from 1 to 5) is colored, dark blue being the lowest value and red the highest. AA, amino acids; IM PQ-9, inner membrane plastoquinone-9; NDH, NADH dehydrogenase complex; OPDA, 12-oxo phytodienoic acid.

As shown by chlorophyll fluorescence (Fig. 2), plastids from vascular cells and mesophylls cells are both chloroplasts. To assess whether these chloroplasts may have different metabolic functions, we performed a TMT-labelled 4-h-long gradient LC-MS proteomic analysis of plastids extracted from both tissues by IPTACT (Supplementary Fig. S4). We plotted the first two components of a principal component analysis from normalized proteomic data, and observed that replicates were widespread on component 1 (42%) (Supplementary Fig. S5). Components 2 and 3, accounting for 19% and 8% of the variance, respectively, did not particularly increase the separation between the tissue types nor group the replicates. These plots revealed that, for both cell types, plastids did not especially differ in their proteomic profiles. More than 1600 proteins were detected in both cell types, with ca. 1300 (80%) predicted to be plastidial (Supplementary Dataset S1). The remaining 20% are predicted to be contaminants mostly from the peroxisomes, Golgi, and cytosol. Also worth noticing, more contaminants were present in the mesophyll extract than in the vascular cell extract, even though we do not have any robust explanation for this. We hypothesized that this may result from the number of tagged plastids, which is much higher in mesophyll than vascular cells (from the same amount of starting material) and which therefore may drag more contaminants during the isolation procedure, yet this remains rather speculative. Among the 1342 proteins predicted to be plastidial, 705 were confirmed plastidial according to SUBAcon 5. Interestingly, 649 proteins were detected at a similar abundance between the two tissues, and only 53 proteins accumulated significantly more in vascular cells while three were found to be significantly more abundant in chloroplasts of mesophyll cells (*P*≤0.05; Fig. 5A). Altogether, the 7.9% of proteins found to be in a significantly differential abundance in the SUBAcon-curated subset are statistically different (*P*=0.0005359344 with Fisher’s exact test) from the 12.2% of proteins found when considering the number of proteins with a significantly different abundance (202) in the total set of proteins for which the TMT isobaric labelling quantification was obtained for each replicate (i.e. 1661).

Interestingly, most of the proteins associated with the primary metabolism of a chloroplast (i.e. photosynthesis, photorespiration, lipid/tetrapyrrole/isoprenoids biosynthesis, etc.) were not differentially regulated between the plastids of the two tissues (Supplementary Fig. S6). Focusing only on the proteins found with a different abundance, we performed a GO term analysis (TAIR10) using g:Profiler and Revigo (Supek *et al.*, 2011; Raudvere *et al.*, 2019). Figure 5B provides a selection of these GO terms for Biological Process associated with chloroplast functions over-represented in either vascular or mesophyll tissues (*n*=56). This approach allowed the identification of biological functions linked to quinone (GO:1901663) and plastoquinone (GO:0010236) biosynthesis, as well as isoprenoid (GO:0006720) and carotenoid (GO:0016116) metabolic process (Fig. 5B; Supplementary Table S3). In more detail, the nature and abundance of these 53 proteins across all biological samples are reported in Fig. 5C. For instance, five components of the ribosome were more abundant in plastids from vascular cells as compared with plastids from mesophyll tissue. Most interestingly, the tocopherol cyclase vitamin E deficient 1 (VTE1), NAD(P)H dehydrogenase C1 (NDC1), fructose-bisphosphate aldolase 2 (FBA2), plastoglobulin of 34 kDa (PGL34) and the kinase ABC1-like kinase 1 (ABC1K1) are all known to be core proteins or associated with the metabolic function of plastoglobuli, i.e. lipid bodies interacting with thylakoids and notably involved in plastoquinone (PQ-9), plastochromanol (PC-8), δ-tocopherol (vitamin E), and phylloquinone (vitamin K1) biosynthetic process and homeostasis. Also, in line with plastoquinone production in the stroma, two enzymes were found in higher abundance, solanesyl diphosphate synthase 1 (SPS1) and the inner membrane enzyme homogentisate prenyltransferase (HST) (Kim *et al.*, 2015). The accumulation of ATAB2 protein suggests that the biogenesis of photosystems I and II (PSI/II) was promoted (Barneche *et al.*, 2006), although no significant accumulation of PsaA, PsaB, PsbA, and PsbD was observed in plastids from vascular cells (Supplementary Dataset S1). Also, PSI biogenesis seemed enhanced as YCF3-interacting protein (CEST), photosystem I subunit G (PsaG), and PYG7 accumulated in plastids from vascular cells. Additionally, several proteins involved in the cyclic electron flow around PSI were found to be more abundant in plastids from vascular cells. For instance, three members (CRR1, PNSL2, and CFM3a splicing NdhB) of the NADH dehydrogenase complex (NDH), as well as the thioredoxin M4 (TRX-M4), described as a regulator of the NDH complex (Courteille *et al.*, 2013) and of the PGR5/PGRL1-dependent pathway (Okegawa and Motohashi, 2020). Two other thioredoxins, TRX-lilium 3 (At1g07700) and TRX-like (At2g37240), were also found to be more abundant in vascular plastids. Finally, several proteins associated with the biosynthesis of JA were found in a larger abundance in plastids from vascular cells than in their counterparts. Indeed, Allene Oxide Cyclase 1 (AOC1) and lipoxygenase (LOX2) are both involved in the synthesis of 12-oxo phytodienoic acid (OPDA), a precursor of JA. Furthermore, the signal recognition particle of 54 kDA (SRP54), translocating AOC1 to the thylakoids, also accumulated in vascular cells, which ultimately influences OPDA production, and in turn, the biosynthesis of JA (Ji *et al.*, 2021).

## Discussion

Here, we report the application of our IPTACT method (Boussardon and Keech, 2023) to isolate plastids from mesophyll and vascular cells. The method derives from our IMTACT method described earlier (Boussardon *et al.*, 2020; Boussardon and Keech, 2022) with the use of a translational fusion of TOC64 containing eGFP and BLRP at the C-terminus, combined with the *UBQ10::BirA* construct (Fig. 1A). The BirA ligase allows the biotinylation of BLRP. Obviously, other outer membrane proteins of the plastid envelope could be used for this method, but we opted for TOC64 as it was shown to be non-essential for effective protein import in Arabidopsis (Aronsson *et al.*, 2007). Hence, a modulation in TOC64 abundance was less likely to impact protein transport into plastids, as well as to affect the growth phenotype of transgenic plants, which we confirmed as no macroscopic phenotype was observed in the IPTACT lines (Fig. 1B). In this article, the TOC64 translational fusion expression was driven by the *pCAB3* and *pSUC2* promoters leading to eGFP localization in mesophyll cells and vascular tissues plastids, respectively (Fig. 2). Electron microscopy and immunoblot analysis of IPTACT samples confirmed the isolation of plastids in approximately 30 min from tissue homogenization to a final pellet with plastids (Figs 3, 4). Furthermore, we show that the isolated material from IPTACT was perfectly suitable for proteomic analysis, which allowed the detection of more than 1600 proteins, among which 1300 were predicted to be plastidial.

Clearly, the quality of IPTACT and LC-MS analysis is highly dependent on the quality of the sample. Even though, contaminants were hardly detectable by western blot, 20% of the proteins detected by LC-MS are predicted to be localized outside of plastids. Thus, non-plastidial proteins should be filtered out of the analysis. As contaminants were mainly found in IPTACT from mesophyll cells, we hypothesized that the number of tagged plastids present in the crude extract influences the purity of the final pellet containing plastids. If the concentration of plastids is too high, plastids might pull-down contaminants when the magnetic field is applied. Also, the nature of plastids should be considered. As shown both in this study and in Cayla *et al.* (2015), the structure and spatial organization of plastids from mesophyll and vascular cells are different. Thus, it is likely that the physical interactions of the organelles with other cytosolic structures, via, for example, the presence of stromules, could influence the ability of plastids to drag contaminants along during the isolation procedure.

Interestingly, the differences of the average size of plastids isolated from mesophyll and vascular cells has been confirmed (Fig. 3). However, if the ca. 5 µm size was confirmed for mesophyll plastids, plastids isolated from *pSUC2* lines were estimated at ca. 4 µm. The *pSUC2* promoter has been defined as specific for companion cells from which plastid size was previously estimated at 3 µm (Truernit and Sauer, 1995; Cayla *et al.*, 2015). This discrepancy suggests that the *pSUC2* promoter not only allowed the isolation of companion cell plastids but also those from other components of the phloem such as phloem parenchyma cells. This hypothesis is supported by *in vivo* confocal observations made on *pSUC2:PP2-A1:GFP* lines and *pSUC2:GFP* lines, where fluorescence was observed in companion cells for the first construct and in companion cells and phloem parenchyma cells for the second (Cayla *et al.*, 2015). Thus, we cannot rule out that the actual proteomic comparison in this work corresponds to a mix of plastids from companion cells, phloem parenchyma cells and other elements of the phloem.

**Fig. 6.**
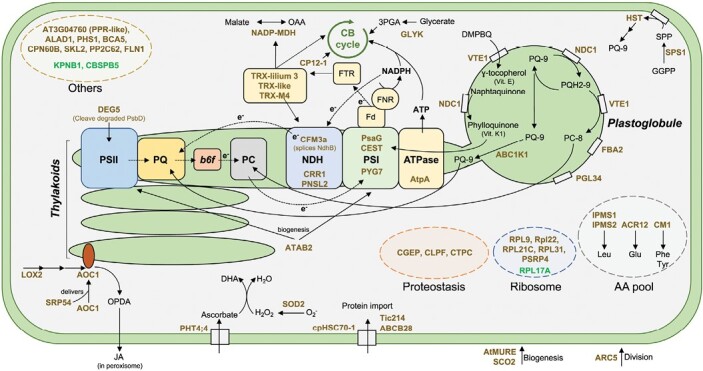
Schematic representation of the differentially abundant plastidial proteins between vascular and mesophyll cells. In vascular tissues, plastids accumulate plastoglobule proteins. Those supply the plastoquinone pool and PSI as they provide PQ-9 and vitamin K1 through the action of NDC1 and ABC1K1. Also, increase in VTE1 could enhance the amount of tocopherol. Plastids from vascular tissues seem to affect preferentially complexes and pools downstream of the PSII. In brown, proteins accumulating in *pSUC2* lines; in green, proteins accumulating in *pCAB3* lines; in black, non-identified products by proteomic; dark green lines, plastid envelope (outer and inner membranes); light green, thylakoids and plastoglobule. *b*_6_*f*, cytochrome *b*_6_*f* complex; Fd, ferredoxin; FTR, ferredoxin–thioredoxin reductase; NDH, NAD(P)H dehydrogenase complex; PC, plastocyanin pool; PQ, plastoquinone pool; PSI, photosystem I; PSII, photosytem II; SPP, solanesyl diphosphate; TRX, thioredoxin.

To our surprise, the proteomic analysis allowed the identification of only 56 proteins differentially accumulated (*P*<0.05) between chloroplasts from the vascular and mesophyll tissues (Figs 5C, 6). None of the primary metabolic functions appeared over- or under-represented, supporting the idea of a ‘conserved’ abundance of proteins dedicated to photosynthesis, photorespiration, metabolism of isoprenoids, tetrapyrroles, fatty acids, etc. per chloroplast, regardless of the tissue it originates from. This also indicates that the pull-down from vascular tissues contains mostly chloroplasts, even though the size distribution was significantly different from the chloroplasts from mesophyll cells. Furthermore, a good confirmation that we successfully enriched plastids from vascular tissue came from the higher abundance of CGEP, a glutamyl peptidase part of the chloroplast proteostasis network. This peptidase, which cleaves after glutamate residues (Bhuiyan *et al.*, 2020), was reported to be in 4-fold higher abundance in chloroplasts of bundle-sheath cells as compared with mesophyll cells in maize (Friso *et al.*, 2010). That said, the vast majority of proteins that were found in a significantly higher abundance were in chloroplasts from vascular cells. Interestingly, a few of metabolic functions seem particularly enhanced in these plastids as detailed here below.

### Amino acid metabolism

The accumulation of Isopropylmalate Synthase 1 (IPMS1), IPMS2, Chorismate Mutase 1 (CM1), and ACT Domain Repeat 12 (ACR12) (Figs 5 and 6) suggests a modulation in the pool of amino acids. Indeed, IPMS1 and IPMS2 are isopropylmalate synthase enzymes known to be key actors in leucine biosynthesis in plastids (de Kraker et al., 2007). CM1 is the first enzyme in the shikimate pathway ultimately leading to the biosynthesis of phenylalanine and tyrosine (Westfall *et al.*, 2014). ACR12 is a protein containing two ACT (named after proteins Aspartate kinase, Chorismate mutase and TyrA) domains similar to the glutamine binding domain (GlnD) of *E. coli*, and has been proposed to be an amino acid sensor in Arabidopsis (Sung *et al.*, 2011). Though still unclear, the function of ACR12 has been associated with glutamate synthesis in roots through the ferredoxin-dependent glutamate synthase (Fd-GOGAT) (Takabayashi *et al.*, 2016), and one could envision that ACR12 accumulates and shares the same function in both subterranean and above ground vascular tissues. In conclusion, plastids from vascular tissues could preferentially promote the biosynthesis of certain amino acids (Fig. 6).

### JA metabolism

It is known that precursors of JA are synthesized in plastids through the action of several enzymes. This results in the production of OPDA, which is then translocated to the peroxisome for the biosynthesis of JA. Here, we observed an accumulation of LOX2, AOC1, and cpSRP54 (Figs 5 and 6). LOX2, a lipoxygenase, and AOC1, one of the four allene oxide cyclases, participate in the catalysation of α-linolenic acid that leads to the synthesis of OPDA. Furthermore, it was recently shown that cpSRP54 allows the translocation of AOCs to the thylakoid membranes (Ji *et al.*, 2021). Interestingly, the turnip mosaic virus P1 sends cpSRP54 to degradation by the 26S proteasome to suppress JA biosynthesis. Once in sieve elements, it is currently hypothesized that viruses use endoplasmic reticulum and membrane-enveloped structures to accumulate to critical levels before being loaded into the phloem sap (Wan *et al.*, 2015; Kloth and Kormelink, 2020). Since our data support the idea that plastids from vascular tissues have a higher rate of the metabolic pathway toward the production of OPDA, and in turn towards the production of JA, one may propose that such a metabolic difference is relevant to hampering the propagation of pathogens (particularly viruses) via the phloem.

### Plastoglobuli and redox turnover

Studies on the plastoglobule proteomes in Arabidopsis have reported about 30 proteins are present (van Wijk and Kessler, 2017). It is likely that the content of a plastoglobule is influenced by the developmental stage, tissue type, and stress level in a given tissue (Liu, 2016; Michel *et al.*, 2021; Arzac *et al.*, 2022). For example, during leaf senescence, the degradation of ­plastoglobuli leads to the conversion of fatty acids into phloem-mobile sucrose (Kaup *et al.*, 2002). Although often defined as lipid droplets, plastoglobuli are also known to be involved in the metabolism of tocopherols, plastoquinones (PQ-9/PQH2-9) and phylloquinone. Interestingly, we identified only five plastoglobule proteins that significantly accumulated in plastids from vascular cells as compared with plastids from mesophyll cells. Yet, the accumulation of VTE1, NCD1, and ABC1K1, as well as of SPS1 and HST, strongly suggests a metabolic switch for a higher redox capacity in the plastids from vascular cells.

Indeed, PQ is formed in the inner membrane of plastids and in plastoglobuli (Fig. 6). It was proposed that a SPS homodimer interacts with fibrilin 5 (although not detected here) in the stroma to release solanesyl diphosphate (not differentially accumulated), further processed by HST in the inner membrane, and which ultimately produces PQ-9 (Kim *et al.*, 2015). In chloroplast, PQ-9/PQH2-9 is an electron carrier associated with the photosynthetic electron flow, but it also acts as an antioxidant, especially as a ^1^O_2_ scavenger (Kruk and Szymanska, 2021). Furthermore, ABC1K1 (also referred to as PROTON GRADIENT REGULATION 6 (PGR6)) modulates plastoquinone homeostasis by regulating the transfer of PQ-9 from plastoglobuli to thylakoids (Lundquist *et al.*, 2013; Pralon *et al.*, 2019) (Fig. 6), thus assisting the photosynthetic electron flow upon changing conditions. Besides proteins affecting the PQ pool, the accumulation of VTE1 suggests a higher level of δ-tocopherol. Tocopherols are well known antioxidants, and it was shown that δ-tocopherol is necessary for phloem loading, as *vte* mutants had a reduced export of photoassimilates, as well as to amass callose onto phloem parenchyma cell walls (Maeda *et al.*, 2006). Finally, and worth noticing, the PHT4;4 protein, an ascorbate transporter located at the chloroplast envelope, also accumulated in plastids from vascular cells as compared with plastids from mesophyll cells (Figs 5 and 6).

Taken together, these results indicate that there is a higher redox capacity in plastids from vascular cells that is reflected in the accumulation of proteins supporting the metabolism of redox mediators such as PQH2, PC-8, tocopherols, and ascorbate, as well as the presence of known redox regulators such as TRX-m4, TRX-lilium 3, TRX-like, and superoxide dismutase (SOD2). In addition, the accumulation of proteins associated with NDH and PSI suggests a higher activity around PSI. It seems unlikely that this indicates an issue with the light harvesting and photosynthesis *per se*, but rather that such a mechanism would contribute to securing the transport of ions to or from the chloroplasts in vascular cells, which is likely under a higher ion strength as compared with mesophyll cells (Lackney and Sjolund, 1991). Indeed, due to its role in the transport of solutes (Pritchard, 2007) as well as a component of signaling in plants (Gaupels *et al.*, 2016; Liesche and Patrick, 2017), the phloem undergoes frequent fluctuations of its redox balance (Herschbach *et al.*, 2010). Consequently, in order for chloroplasts to retain their functionality under these challenging conditions, we propose that they deal with this situation by activating redox balancing pathways around PSI. One plausible explanation for this would be that PSI is much more capable of dealing with higher energy electrons than PSII due to the presence of alternative electron transport shuttles such as the cyclic electron flow or Mehler reaction, for example (Hüner et al., 2012). Furthermore, an enhanced cyclic electron flow would also favor ATP production over the reduction of NADP^+^, which may become relevant for supporting the active pumping of ions across the chloroplast membrane, and thus compensating for the higher ion strength encountered in vascular cells.

To conclude, our work demonstrates the technical feasibility of isolating plastids in a tissue-specific manner, which paves the way to deepening our understanding of metabolic functions associated with the different types of plastids. For example, our work here provides strong evidence that plastids from vascular tissue have a higher redox turnover to secure an optimal functioning under the high solute strength encountered in vascular cells, which are under constantly fluctuating ion strength. More generally, IPTACT also opens new perspectives in the study of tissue-specific post-transcriptional regulation in Arabidopsis under normal or stress conditions. In fact, investigations on mutants with defective RNA processing showed that RNA editing, splicing, and stability were important regulators of the plant stress response. For example, mutants of RNA editing factors led to a hypersensitivity to cold stress in both Arabidopsis and rice (Wang *et al.*, 2016; Cui *et al.*, 2019). Also, the weak allele of the DEAD-box RNA helicase *RH3* mutant, regulating five splicing sites, induced a lower tolerance to salt stress (Gu *et al.*, 2014). Interestingly, evidence shows that RNA processing is tissue- and organ-dependent while post-transcriptional modifications are generally assessed in the whole leaf, and hence lack tissue-specific resolution (Tian *et al.*, 2019). Hence, data generated with IPTACT could provide fundamental results on the tissue-specific regulation of plastid gene expression and how it impacts metabolic functions, such as photosynthesis, photorespiration, or fatty acid and isoprenoid biosynthesis, which are essential for plants fitness particularly under challenging growth conditions.

## Supplementary data

The following supplementary data are available at *JXB* online.

Dataset S1. Proteomic data from mass spectrometry.

Dataset S2. Compiled raw data presented in this study.

Fig. S1. Chlorophyll content of IPTACT plants.

Fig. S2. Protein quantification from IPTACT.

Fig. S3. Complete western blots membranes of plastids isolated by IPTACT.

Fig. S4. Immunoblot controls of plastids purified by IPTACT prior proteomic analysis.

Fig. S5. Principal component analysis of vascular and mesophyll plastids.

Fig. S6. MapMan analysis of changes between mesophyll and vascular plastid proteomes.

Table S1. Primers used in this study.

Table S2. Protein quantification.

Table S3. GO terms (Biological Process).

erad133_suppl_Supplementary_Figures_S1-S6

erad133_suppl_Supplementary_Tables_S1-S3

erad133_suppl_Supplementary_Dataset_S1

erad133_suppl_Supplementary_Dataset_S2

## Data Availability

All data are available in the article and its supplementary data. Constructs are available upon request to the corresponding author (OK).
