# Supplementary material for: Comparison of plastid proteomes points towards a higher plastidial redox turnover in vascular tissues than in mesophyll cells
Source: J Exp Bot. 2023 Apr 7;74(14):4110–24. doi: 10.1093/jxb/erad133 (PMC10400147; doi:10.1093/jxb/erad133)
Supplement: erad133_suppl_Supplementary_Figures_S1-S6 [file erad133_suppl_supplementary_figures_s1-s6.pdf]

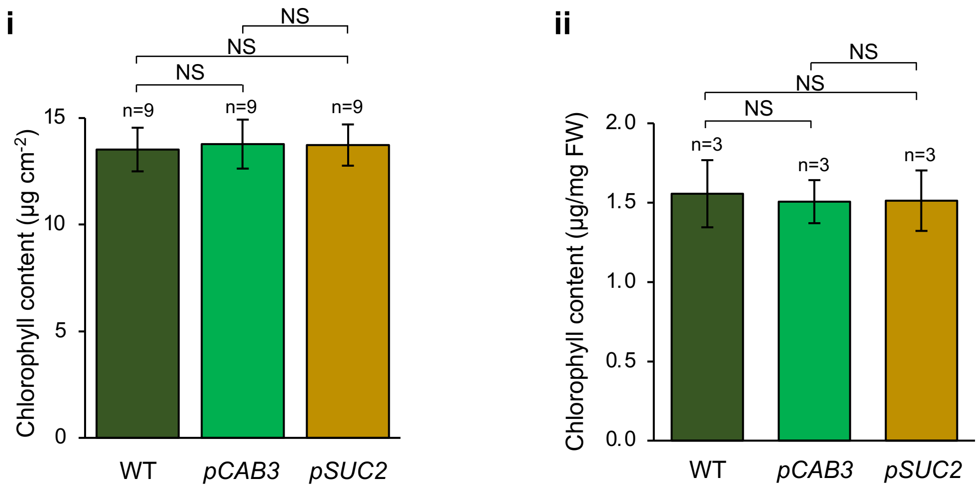

Figure S1. Chlorophyll content of IPTACT plants. Chlorophyll content was obtained using (i) a Dualex 4 scientifc apparatus or (ii) an acetone extraction. Measures were taken on 6-week-old plants. (i) Three plants per genotype and three leaves per plant were used for statistical analysis (Student's t-test; two sided; n=9 biologically independent replicates; NS=not significant). (ii) Three plants per genotype and one leaf collected per plant were used for statistical analysis (Student's t-test; two sided; n=3 biologically independent replicates; NS=not significant).

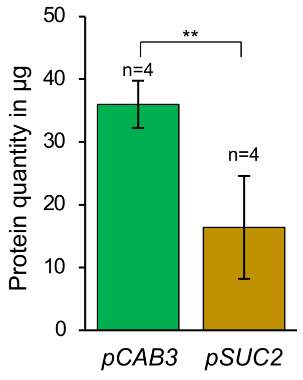

Figure S2. Protein quantification from IPTACT. Proteins obtained from plastids isolated by IPTACT were quantified by Bradford. For each replicate (n=4), IPTACT was performed using 1 g of leaves and 30 µl of beads. Results are mean. Error bars represent the standard deviation; a Student's t-test (two sided; n=4 biologically independent replicates) showed statistically significant differences at ** p<0.01.

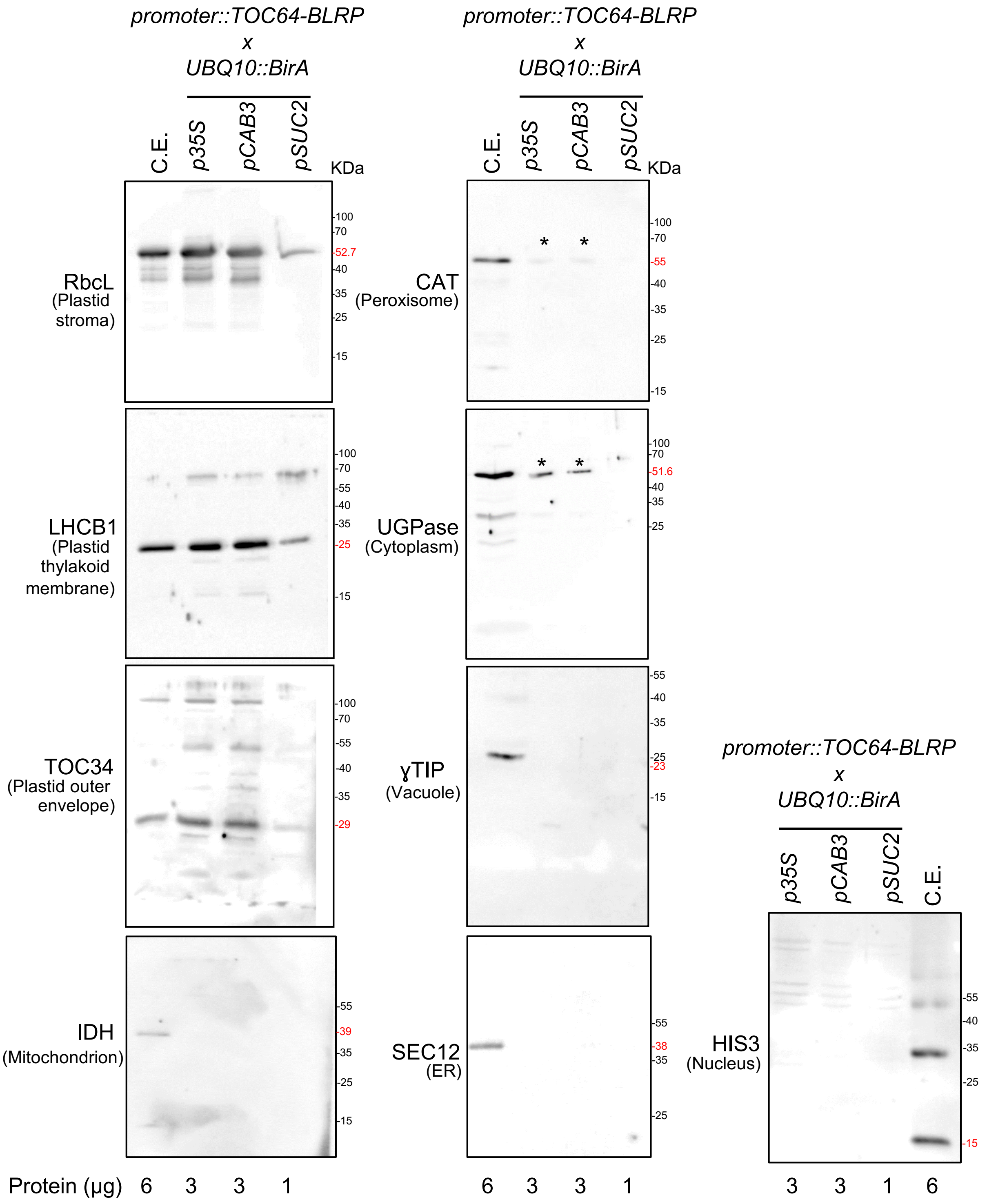

Figure S3. Complete western blots membranes of plastids isolated by IPTACT. This figure shows the full membranes used in Figure 4. On the same membrane, different sub-compartment markers are detected in a 35S crude extract (C.E.) and *35S*-, *pSUC2*-, *pCAB3*-IPTACT extracts. Stars (*) represent traces of peroxisomal and cytoplasmic contaminations. For a single antibody, pictures were taken on the same membrane. In red: expected molecular weight in kilodaldon (kDa) of the protein of interest. The lanes corresponding to 35S crude extract and *35S*-IPTACT are published and described in detail in Boussardon and Keech (2023).

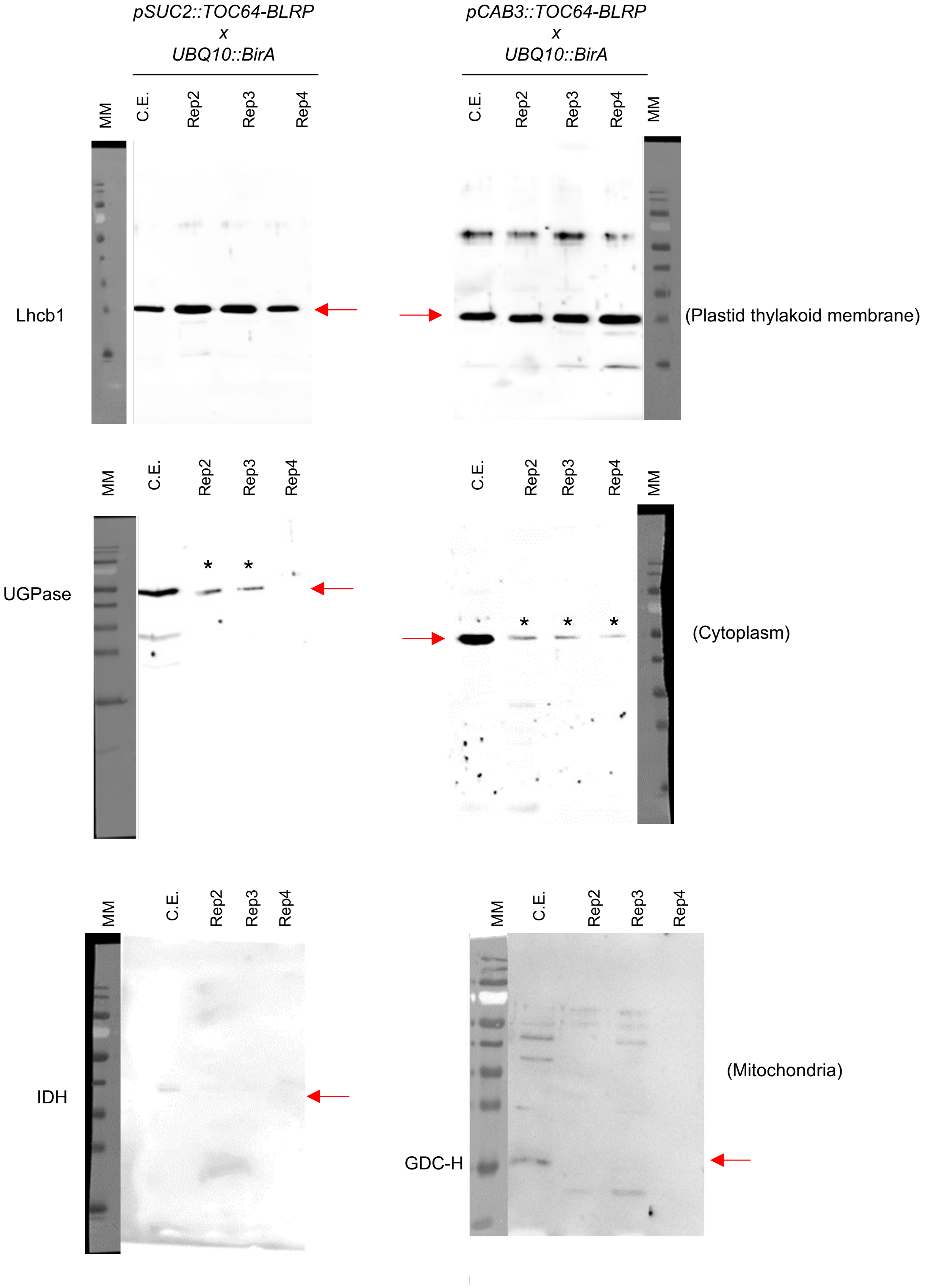

Figure S4. Immunoblot controls of plastids purified by IPTACT prior proteomic analysis. 2 µg of crude extract (C.E) and purified biotinylated plastids from shoots were immunoblotted with anti-Lhcb1 to observe plastids enrichment, anti-IDH or anti GDC-H and anti-UGPase were used to check contamination in mitochondrial and cytoplasmic proteins, respectively. Stars (*) represent traces of cytoplasmic contaminations. MM: molecular marker. Red arrows point toward the expected size of the protein.

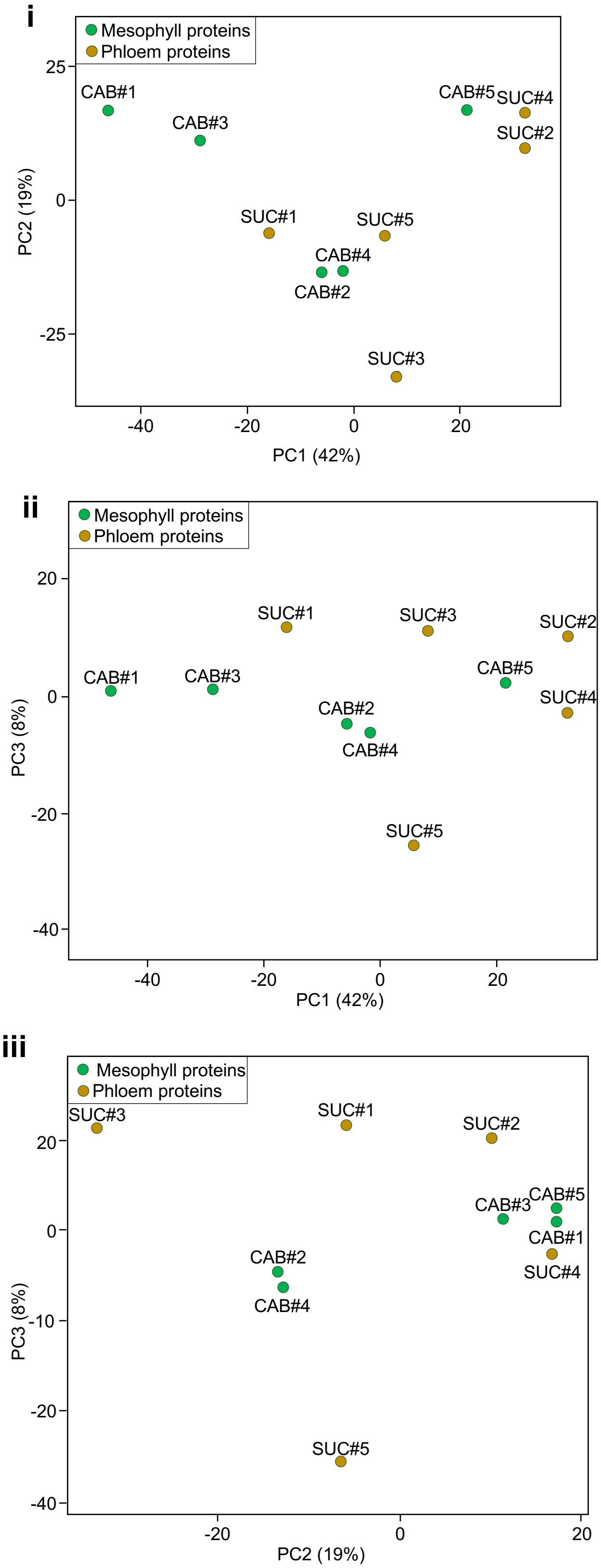

Figure S5. Principal component analysis of vascular and mesophyll plastids. Resemblances and variations within the protein samples were observed by PCA; first component (42%), second component (19%) and third component (8%). (i) PC1 vs PC2; (ii) PC1 vs PC3; (iii) PC2 vs PC3.

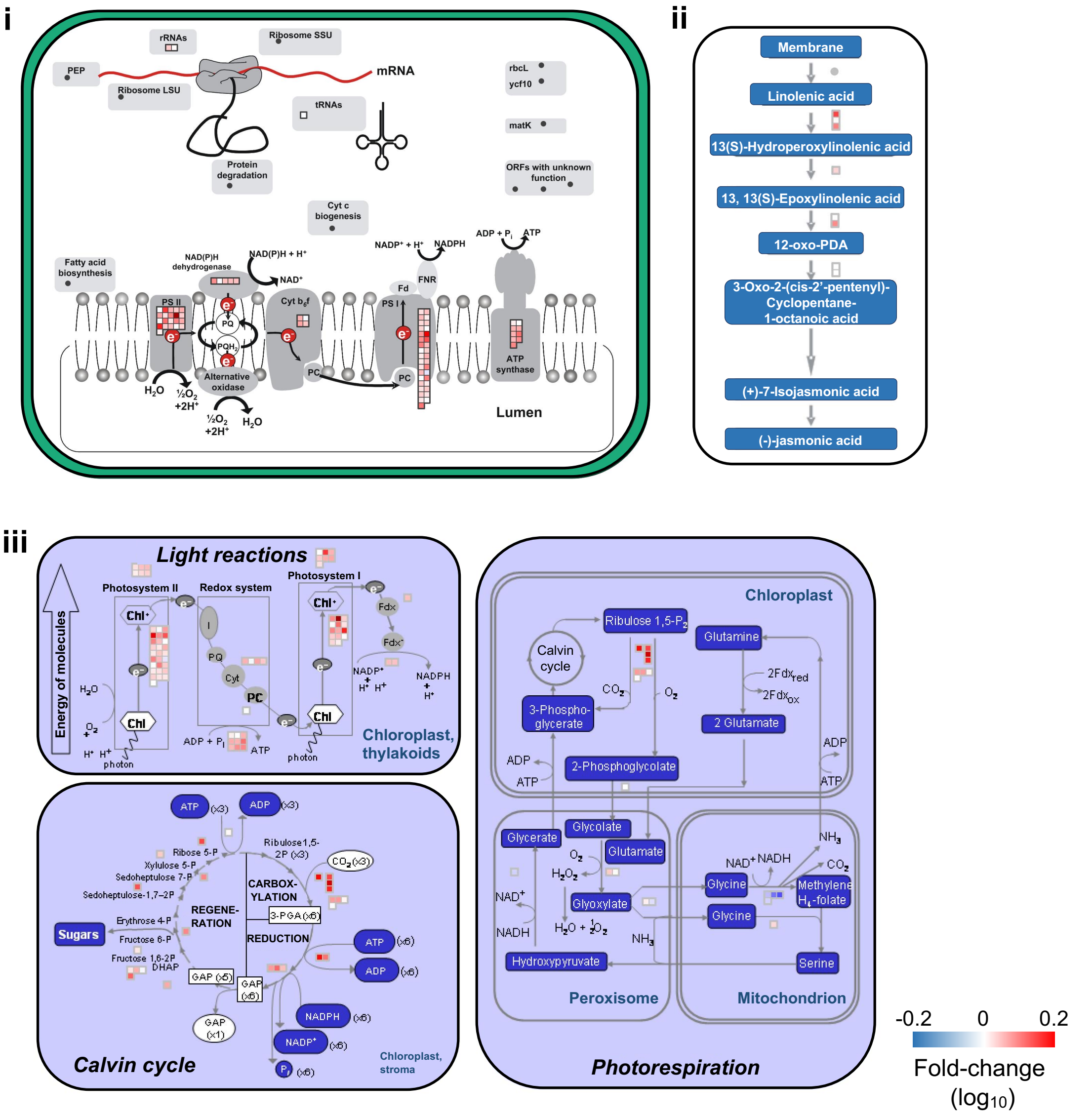

Figure S6. MapMan analysis of changes between mesophyll and vascular plastid proteomes. The 1672 proteins detected were mapped to the MapMan Ath_AGI_LOCUS_TAIR10_Aug2012.m02 (i) ChloroPlast_CustomArray mapping (77 data points visible); (ii) JA synthesis mapping (8 data points visible); (iii) Photosynthesis mapping (116 data points visible). Red and blue squares represent increased fold-change (transformed into $\log_{10}$) in vascular or mesophyll tissues, respectively.
